# Temperature stress differentially modulates transcription in meiotic anthers of heat-tolerant and heat-sensitive tomato plants

**DOI:** 10.1186/1471-2164-12-384

**Published:** 2011-07-31

**Authors:** Craita E Bita, Sara Zenoni, Wim H Vriezen, Celestina Mariani, Mario Pezzotti, Tom Gerats

**Affiliations:** 1 Radboud University, IWWR, Plant Cell Biology and Plant Genetics, Graduate School of Experimental Plant Sciences, Heyendaalseweg 135, 6525AJ, Nijmegen, The Netherlands; 2 Dipartimento di Biotecnologie, Universitá degli Studi di Verona, Italy; 3 Nunhems Netherlands B.V., P.O. Box 4005, 6080 AA Haelen, Netherlands

## Abstract

**Background:**

Fluctuations in temperature occur naturally during plant growth and reproduction. However, in the hot summers this variation may become stressful and damaging for the molecular mechanisms involved in proper cell growth, impairing thus plant development and particularly fruit-set in many crop plants. Tolerance to such a stress can be achieved by constitutive gene expression or by rapid changes in gene expression, which ultimately leads to protection against thermal damage. We have used cDNA-AFLP and microarray analyses to compare the early response of the tomato meiotic anther transcriptome to moderate heat stress conditions (32°C) in a heat-tolerant and a heat-sensitive tomato genotype. In the light of the expected global temperature increases, elucidating such protective mechanisms and identifying candidate tolerance genes can be used to improve breeding strategies for crop tolerance to heat stress.

**Results:**

The cDNA-AFLP analysis shows that 30 h of moderate heat stress (MHS) alter the expression of approximately 1% of the studied transcript-derived fragments in a heat-sensitive genotype. The major effect is gene down-regulation after the first 2 h of stress. The microarray analysis subsequently applied to elucidate early responses of a heat-tolerant and a heat-sensitive tomato genotype, also shows about 1% of the genes having significant changes in expression after the 2 h of stress. The tolerant genotype not only reacts with moderate transcriptomic changes but also exhibits constitutively higher expression levels of genes involved in protection and thermotolerance.

**Conclusion:**

In contrast to the heat-sensitive genotype, the heat-tolerant genotype exhibits moderate transcriptional changes under moderate heat stress. Moreover, the heat-tolerant genotype also shows a different constitutive gene expression profile compared to the heat-sensitive genotype, indicating genetic differences in adaptation to increased temperatures. In the heat-tolerant genotype, the majority of changes in gene expression is represented by up-regulation, while in the heat-sensitive genotype there is a general trend to down-regulate gene expression upon MHS. The putative functions associated with the genes identified by cDNA-AFLP or microarray indicate the involvement of heat shock, metabolism, antioxidant and development pathways. Based on the observed differences in response to MHS and on literature sources, we identified a number of candidate transcripts involved in heat-tolerance.

## Background

Abiotic stresses such as extreme temperatures, drought, flooding or chemical toxicity, pose serious threats to agricultural production. A rapid adaptation or an innate tolerance mechanism can protected the further development of the plant and importantly for yield, secure successful fruit-set. Gamete development in angiosperms takes place within two floral organs; the male stamen and the female pistil [1,2]. The male gamete development starts with the differentiation of the reproductive tissues of the anther. After meiosis of the pollen mother cell and mitotic divisions, microspore maturation follows, resulting in the mature pollen grain. After initiation, highly specialized anther tissues will acquire non-reproductive (e.g., the tapetum for support, stomium for dehiscence) or reproductive functions (pollen - formation). Both tapetum and microspore development are essential for male fertility as documented by numerous studies on male sterile mutants [3-9]. In Petunia, a cDNA-AFLP study revealed that during meiosis in anthers, under standard conditions, besides the typical meiotic genes, other genes are also important for the development of the pollen grains. Thus, during meiosis, tapetum differentiation genes, serine-proteinases, hormone metabolism genes, genes involved in cell wall biosynthesis and even ribonucleases and polyamine biosynthesis are all modulated in expression [10]. These genes represent members of physiological and metabolic pathways that are naturally differentially expressed during pollen biogenesis under normal conditions.

Tomato (*Solanum lycopersicum *L.) displays marked responses to heat, similar to other crop species including pepper, potato, melon, cowpea, wheat, common bean, rice and barley [11-13]. Hot summers in many agricultural regions can negatively affect the vegetative and reproductive growth phases of such crops [14] and can result in up to 70% losses in tomato yield [15]. However, heat stress has numerous specific effects depending on the genotype. Physiological observations both under field and greenhouse conditions show a variable degree of tolerance between different genotypes. A wide range of heat stress phenotypes has been described [16,17]. For example, of five tomato cultivars grown under moderate heat stress (MHS) conditions (32°C day and 26°C night), only one set fruit [18]. The differences in pollen grain development among the tolerant genotypes are most critical factors to determine fruit set under heat stress. Comparing the effects of heat stress on a heat-tolerant and a heat-sensitive tomato cultivar showed that temperature stress affected mainly the development of pollen grains, where reduced viability was more pronounced in the heat-sensitive cultivar [19].

Under heat stress, it is the pollen grain development and particularly meiosis that shows the highest susceptibility, followed by germination and pollen tube growth, which in more severe cases is also significantly affected [20-22]. Recently, an analysis of maturing tomato microspores (mitotic anthers) exposed to heat-shock conditions has shown no differential gene expression between heat-tolerant and heat-sensitive genotypes but it seems that the capacity for thermotolerance may be achieved by modulating the expression levels of such 'responsive' genes prior to heat stress exposure [23].

In this paper we present an analysis of the different changes in gene expression in developing meiotic anthers (the first and most sensitive anther developmental stage) in response to MHS of tolerant and sensitive tomato genotypes. Assuming the protective mechanisms are initiated shortly after the commencement of the stress period, we decided to study gene expression changes in the first hours of MHS. Both cDNA-AFLP and Combimatrix microarray technology were applied to obtain a general overview of molecular mechanisms that participate in the response to MHS of anthers from a heat-tolerant and two heat-sensitive genotypes of tomato. We describe a set of candidate genes and pathways that open up the possibility of understanding and modulating male heat tolerance in tomato and other commercially important *Solanaceae *crops.

## Results and discussion

In order to analyse the dynamics of transcriptional responses to moderate heat stress (MHS) in meiotic anthers of tolerant and sensitive genotypes, the temperature range for the experiment was chosen based on agronomically relevant temperatures shown to have a significant effect on pollen grain viability [24], rather than using classical heat shock conditions of 42°-45°C [23]. We focused our analysis of gene expression on whole meiotic anther cones isolated from flower buds of plants that were exposed to MHS (32°C/26°C, day/night) for up to 30 h, and compared this to standard temperatures (26°C/18°C day/night). To analyse the heat stress response we used several genotypes characterised by plant breeders as relatively tolerant and sensitive.

### Identification of differentially expressed transcripts during MHS by cDNA-AFLP

**Figure 1:**
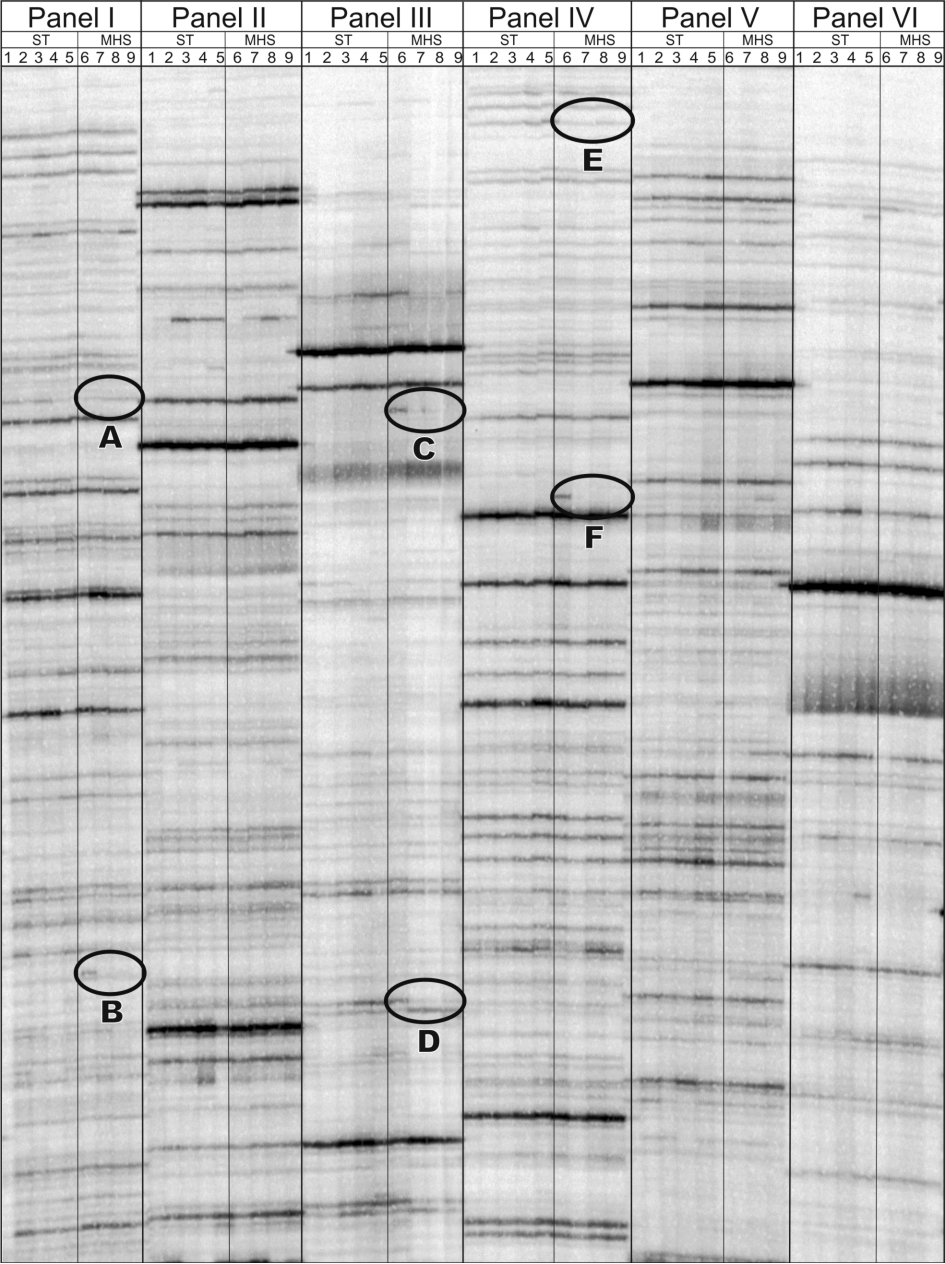
**cDNA-AFLP gene expression profiling**. Example of cDNA-AFLP profiling. Bands of interest are shown with circles. Panels I through VI represent individual cDNA-AFLP primer combinations. TDFs are displayed as bands over 5 different timpoints (1-5) under standard conditions (ST) and under MHS. Various different forms of differentially displayed fragments are shown (A through F).

The aim of this experiment was to investigate the temporal impact of MHS on gene transcription in meiotic anthers in tomato. In order to establish the most critical time points when responses to MHS are initiated and therefore a focus point for a further analysis, we used a heat-sensitive tomato cultivar (own observations) Moneymaker (MM) and recorded the number, the type of genes affected, and the timing of the transcriptional response. Total RNA was extracted from whole meiotic anther cones. To provide a rapid view on gene expression profiles, a modified cDNA-AFLP protocol was used [25]. Figure 1. shows an example of cDNA-AFLP profiling with up and down regulation of genes as a result of MHS. In total 92 cDNA-AFLP primer combinations were performed and this screen lead to the identification of approximately 7300 independent transcript-derived fragments (TDFs) of 100-500 bp. Using the method described previously [10], we estimate that around 30% of the transcriptome of meiotic anthers has been visualised in this screen. The cDNA-AFLP allows an unbiased screening of the genome and is not restricted to genes that are available in the public databases as is the case for micro array analysis.

The most marked transcriptional responses for the heat-sensitive genotype were found at 2 h after initiating MHS: 96 TDFs showed a clear differential expression pattern. Of these, 41 showed a down-regulated pattern, 26 TDFs were transiently induced, 16 were up-regulated and 13 were transiently repressed. No further significant changes in gene expression were found after 6, 16 or 30 h of MHS. As a next step, a selection of TDFs exhibiting differential gene expression were sequenced in order to gain insight into the processes involved in the response(s) to MHS.

From the total set of displayed TDFs, 25 were sequenced based on their potentially interesting differential expression profiles over time. Table 1 summarises the expression patterns of these TDFs and provides the homology-based putative functions of the associated genes. Using the tomato Gene Index (DFCI), 14 TDFs showed sequence similarity to stress-related genes not only from tomato but also other plant species. After submission to Uniprot databases, these homologies suggested the involvement of metabolism, transport, heat shock as well as oxido-reductive processes. Another 11 fragments showed no homology with known genes. This is likely to be due to the small size of the sequenced fragments and the bias for 3'-UTR in the cDNA-AFLP method used [24]. The 25 identified transcripts all showed 100% identical to tomato ESTs (DFCI) and the majority show 25% to 55% similarity (nucleotide level) with characterized transcripts from other species. All data regarding the identified transcripts can be found in the online public GEO database. The rapid change in transcriptional modulation after the initiation of heat stress suggests a short time-window to initiate protection and detoxification mechanisms.

**Table 1:** Characterisation of differentially expressed TDFs in MM from the cDNA-AFLP analysis

TC number and homology		e-value and putative function
1	TC172981 similar to DNA-binding PD1-like protein; *Pisum sativum*, partial (35%)	6.2 e-36 metabolism

2	TC211882 similar to 70 kD heat shock protein; *Arabidopsis thaliana *partial (25%)	7.1 e-32 heat shock response

3	TC197024 homologue to cytochrome c oxidase subunit 1; *Amoebidium* *parasiticum *partial (3%)	1.1 e-13 oxidation reduction

4	TC213206 homologue to calcium/calmodulin-dependent protein kinase CaMK3; *Nicotiana tabacum*, partial (47%)	1.0 e-209 heat shock response

5	AM821191 similar to putative peptide transporter; *Arabidopsis thaliana*, partial (13%)	7.9 e-81 transport

6	TC204777 similar to amino acid transporter-like protein; *Arabidopsis* *thaliana*, partial (57%)	2.7 e-32 transport

7	TC200797 similar to 4Fe-4S ferredoxin iron-sulfur binding domain protein; *Alkalilimnicola ehrlichel*, partial (7%)	9.0 e-0.7 transport

8	TC191544 Phosphosulfolactate synthase-related protein; *Solanum lycopersicum*, complete	4.8 e-22 heat shock response

9	TC495251 similar to OSJNBb0015D13.13 protein; *Oryza sativa*, partial (87%)	0.996 Metabolism, cell wall

10	TC209114 homologue to 40S ribosomal protein S8-like protein; *Solanum* *tuberosum*, complete	2.6 e-28 translation

11	BM066544 weakly similar to TGF-beta receptor type l/lI extracellular region; *Medicago truncatula*, partial (8%)	2.4 e-41 transport

12	TC197929 similar to uncharacterized protein; *Vitis vinifera *partial (44%)	8.8 e-12 unknown

13	BI209494 similar to Os02g0159700 protein; *Oryza sativa *Japonica Group, partial (44%)	8.6 e-08 oxidation reduction

14	TC208178 similar to P70 protein; *Nicotiana tabacum* partial (42%)	3.6 e-12 heat shock response

15	TC208223 Similar to cytosolic ascorbate peroxidase 1; *Solanum* *lycopersicum*, partial (32%)	1.1 e-19 oxidation reduction

16	TC210545 similar to 3-deoxy-D-arabino-heptulosonate 7-phosphate synthase; *Vitis vinifera*, partial (66%)	0.26 metabolism

17	EL492476 homologue to succinyl-CoA ligase alpha 1 subunit; *Solanum* *lycopersicum*, partial (7%)	0.992 metabolism

18	TC203685 similar to DnaJ protein; *Arabidopsis thaliana*, partial (24%)	1.6 e-19 heat shock response

19	TC198815 weakly similar to RNA polymerase Rpb4; *Medicago truncatula*, partial (52%)	2.3 e-38 translation

20	TC193737 homologue to ribosomal protein S6-like protein; *Solanum* *tuberosum*, complete	3.6 e-41 Translation

21	TC197647 similar to elicitor-inducible protein EIG-J7; *Capsicum annuum*, partial (84%)	6.0e-13 stress response

22	TC200797 similar to 4Fe-4S ferredoxin iron-sulfur binding domain protein; *Alkalilimnicola ehrlichei*, partial (7%)	2.8 e-34 transport

23	EB174193 similar to chromosome chrl scaffold_5 whole genome shotgun sequence; *Wis vinifera*, Rep:, partial (13%)	0.048 unknown

24	BY840013 Similar to *Zea mays *18S ribosomal RNA gene partial sequence, partial (20%)	3.1 e-80 translation

25	TC213983 similar to chromosome chr15 scaffold_40 whole genome shotgun sequence; *Vitis vinifera*, partial (10%)	3.1 e-27 unknown

### Validation of cDNA-AFLP expression patterns

**Figure 2:**
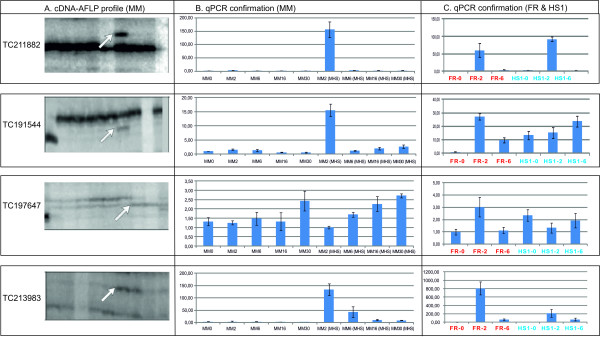
**Validation of the cDNA-AFLP profiling with q-PCR**. Four TDFs (Panel A) corresponding to TC211882, TC191544, TC197647 and TC213983 were verified with quantitative q-PCR in the genotype Money Maker (MM; Panel B) at time points 0 h, 2 h, 6 h, 16 h and 30 h under standard growth and MHS conditions. To further analyze the expression of the same genes in the heat-sensitive (FR) and heat-tolerant (HS1) genotypes, q-PCRs were also carried out on RNA from FR and HS1, for the time points 0 h, 2 h and 6 h of MHS (Panel C). The bars represent expression values derived from the delta CT values and error bars are given as standard deviation.

In order to confirm the observed cDNA-AFLP expression profiles and also to link these to the expression patterns found in the microarray experiments described below, quantitative RT-PCR (q-PCR) experiments were carried out. The expression pattern of 4 TDFs from different functional classes was confirmed by q-PCR in the heat-sensitive genotype MM. Additional confirmation and correlation between the cDNA-AFLP and microarray data were obtained by performing q-PCR in the two genotypes used for the microarray analysis, HS1 (Heat Set 1; heat-tolerant) and FR (Falco Rosso; heat-sensitive) (Figure 2). The q-PCR showed that transcripts similar to a 70 kD heat shock protein (TC211882), the heat shock protein Hsa32 (TC191544), an elicitor induced protein (TC197647) and an uncharacterised TDF (TC213983) have similar expression patterns in the two sensitive genotypes and interestingly, contrasting expression profiles in the tolerant genotype (Figure 2).

We conclude that the cDNA-AFLP experiments showed the effect of MHS on the meiotic anther transcriptome to be visible as of 2 h of treatment with different effects in heat sensitive and tolerant genotypes. In response to MHS, a differential modulation of approximately 1% of the anther transcriptome was observed in the MM genotype. In an effort to identify components involved in the heat stress response, TDFs showing differential gene expression during the heat treatment have been sequenced and analysed further. The identified genes are significantly changed in expression and indicate that meiotic anther development is sensitive to heat stress as early as 2 h of MHS.

### Microarray analysis complements cDNA-AFLP analysis and indicates differential responses to heat stress in heat-tolerant and heat-sensitive genotypes

**Figure 3:**
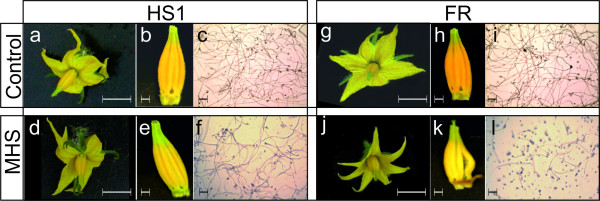
**Tomato phenotypes under heat stress**. Comparison of flower and anther development under control and MHS conditions (two weeks) in the tolerant genotype HS1 and the sensitive FR. Panels a, d, g, j: whole flowers; panels b, e, h and k: isolated anther cones; panels c, f, i and l: germinating pollen. Size bars represent 10 and 3 mm respectively.

Since cDNA-AFLP demonstrated changes in gene expression in the MM tomato genotype early on during MHS, we chose an early time frame (0, 2 and 6 h) to compare the transcriptome of meiotic anthers exposed to MHS for two contrasting genotypes; HS1, a heat-tolerant hybrid that sets fruit at temperatures as high as 38°C in the field and FR, a relative heat-sensitive hybrid. Given the current global climate change projections [26], plants that can trigger molecular mechanisms to prevent heat damage or exhibit constitutively high levels of heat tolerance are very likely to become more important for agriculture [27]. The effects of environmental factors are often seen in gene expression changes, as predominantly down-regulation of all gene programs for sensitive genotypes or in the case of tolerant genotypes as up-regulation of protective mechanisms [23,28]. Figure 3 shows the phenotype of flowers, anthers and pollen viability of HS1 and FR grown under control conditions and two weeks of MHS. While the flower morphology and pollen germination remain largely unaffected by MHS in the HS1 genotype (91% in the control and 73% under MHS), in FR MHS results in slightly smaller flowers with malformed anther cones and significantly reduced pollen germination (74% in the control and only 22% under MHS).

In tomato, cDNA-AFLP and microarray technology have been previously used to dissect stress responses and already enabled identification of candidate genes for tolerance to both abiotic and biotic stresses such as high temperature, low temperature, salt stress and pathogen attack [23,29-31]. We used the Combimatrix Tomato Array 1.0 to compare and analyse the response to heat in a tolerant and in a sensitive genotype. Modulation of gene expression in the two genotypes was analysed using multiclass, paired, and un-paired Significance Analysis of Microarray; SAM [32].

To evaluate the relationship between genome-wide expression profiles of the two genotypes in relation to the heat treatment, SAM multiclass analysis was performed for three consecutive time points, 0 h-2 h-6 h (Additional file 1, SAM multiclass). Using this statistical approach we were able to identify 56 genes in HS1 and 75 genes in FR whose mean expression was significantly changed across 2 time points or over the entire duration of the heat treatment, at a false discovery rate (FDR) of 5%. Among these genes, 45 are shared between the two genotypes. Transient up-regulation at the 2 h time point in both genotypes was the most common profile, and comparable to that of cDNA-AFLP experiment. Interestingly, up-regulation showed a marked genotypic difference with 12 genes up-regulated in HS1, in comparison to only 5 in FR. Down-regulation of gene expression was the second most frequently observed modulation for FR (16 genes) but not for HS1 (9 genes). These modulation types also show a good correlation with the cDNA-AFLP data, where the sensitive MM genotype is also characterised by a considerable down-regulation of gene expression. These results suggest that the difference in tolerance to MHS between the two genotypes is mainly associated with a moderated transcriptomic response in the heat-tolerant genotype with respect to the number of differentially expressed genes and the level of expression changes.

A further analysis of the heat response per genotype was achieved by looking at differentially expressed genes between three pair wise comparisons with a SAM paired analysis: t0-t2, t2-t6, and t0-t6 (Additional file 1, SAM paired). The statistical nature of the SAM paired analysis, in which samples are assigned to one group and there is also a one-to-one pairing between biological replicates, reveals additional information on subtle differences in the response to heat between the two genotypes, particularly in the sensitive genotype. We detected 74 genes in HS1 and 137 in FR, which exhibited significant changes in expression (FDR = 5%). As in the multiclass analysis, the majority were significantly different for the 2 h time point (t0-t2) with 38 genes significantly changed in HS1 compared to 72 genes in FR. The SAM paired analysis also shows that, in the heat-tolerant HS1 genotype, less transcriptional changes are invested into reacting to the heat stress than in the heat-sensitive genotype FR. Overall, the paired analysis shows that HS1 up-regulates 56 and down-regulates 49 genes, while FR up-regulates 72 genes and down-regulates 109, clearly more when compared to the number of down-regulated genes in the heat-tolerant genotype. The SAM paired analysis allows a more in-depth analysis compared to the SAM multiclass analysis, suggesting not only that the difference in tolerance between the two genotypes is indeed associated with a lower transcriptional response of the heat-tolerant genotype but also with more functional classes and members affected in the sensitive genotype.

To detect if significant differences can also be found between the two genotypes prior to the heat treatment, we used the unpaired SAM analysis (Additional file 1, SAM unpaired). By using this method, where biological replicates are not paired each other between two samples, 24 genes were identified as significantly different between HS1 and FR (FDR = 5%). Of these, 4 are significantly higher in the heat-tolerant genotype, while the other 20 genes are at significantly higher levels in the heat-sensitive genotype. The 4 genes more highly expressed in the tolerant genotype and some of the other 20 more highly expressed in the sensitive genotype are further discussed below with regards to their functional classification. A previous study in salt cress proposed that the salt tolerant plant had a smaller number of salt-regulated genes because of the constitutive high level of expression of stress protection-related genes even under standard growth conditions [33]. Similarly, Frank et al. [23] showed that constitutive expression of a heat shock transcription factor and several heat shock proteins mark these genes as candidates for taking part in microspore thermotolerance. In our case the genes with constitutively higher expression in the tolerant genotype are the heat shock Hsp82 (TC170030), a gene coding for mitochondrial small heat shock protein (msHsp) LeMtHSP (TC187014), a cathepsin B-like cysteine proteinase (TC171192) and the fructose-1,6-bisphosphate aldolase gene (TC176475). We suggest that in HS1, the differentially expressed genes prior to heat treatment may represent a constitutive tolerance to MHS.

The statistical analysis of gene expression during MHS reveals a differential response to heat in the two genotypes, in terms of number of significantly differentially expressed transcripts, their levels of expression and the functional classes they belong to. Moreover, the analysis also shows that significant differences in gene expression are present prior to the commencement of the MHS treatment.

Unlike the microarray analysis, the cDNA-AFLP identified genes involved in the response to heat, which may be interesting as candidates for MHS tolerance. Furthermore, of the 25 sequenced and identified TDFs, 19 were also present on the microarray and 3 even appeared as significantly changed upon MHS on the microarray (TC170123/TC211882; a hsp70 like fragment, TC169993/TC191544; the hsa32 also involved in heat shock response and TC179740/TC213983; a fragment of unknown function). The alternate TC numbering arose from the updated Gene Index Release version 8 to version 11 by the DFCI database (included here for clarity).

### Main components determining gene expression modulation under MHS

**Figure 4:**
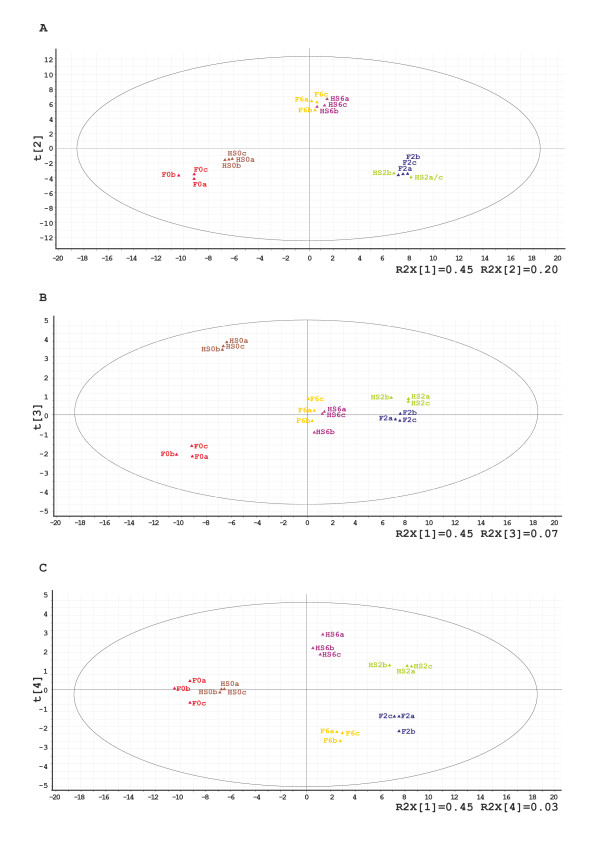
**O2PLS analysis of the components of variation in gene expression in the microarray experiment**. The main components of the changes in gene expression in response to MHS were determined using an O2PLS analysis with SIMCA P+. The plots of predictive component (T2, T3 and T4) versus orthogonal component 1 (T1) are presented. HS1-0a, b and c, HS1-2a, b and c, HS1-6a, b and c, FR-0a, b and c, FR-2a, b and c and FR-6a, b and c represent the three biological replicates (a, b and c) for each experimental time-point (0, 2 and 6) for each genotype (HS1 and FR). Each component clearly discriminates between the two genotypes.

To identify the main components determining the changes in gene expression in response to MHS, an O2PLS (bidirectional orthogonal projection to latent structures) multivariate regression analysis of the data set was done, using the set of 95 significantly differentially expressed genes obtained by multiclass SAM analysis. This technique is well-suited for noisy and correlated variables and obtains robust classification models, having a clear interpretation of the systematic variation useful to characterize each component [33,34]. The two variables chosen were: as X the 95 transcripts, and as Y, the 6 comparison classes (HS0, F0, HS2, F2, HS6 and F6). The OPLS shows four major components of variation (Figure 4). Component 1 in T1/T2 plot separates the three time points distinctly (Figure 4A); in particular T1 separates time 0 from 2 and 6. This result confirms that there is a rapid gene expression response to MHS. In addition, the O2PLS analysis shows a good clustering of the biological repeats in both genotypes. Component 3 in the T1/T3 plot (Figure 4B) separates the HS1 and FR genotypes at 0 h time point and also at 2 h time point. This indicates that prior to the MHS treatment, there is a clear difference in gene expression in the meiotic anthers of these two genotypes. Component 4 in the T1/T4 plot indicates a genotypic difference in the response to the increased period of treatment (Figure 4C). We conclude from this analysis that the major component of variation in transcriptional changes is based on the innate genotypic differences between HS1 and FR prior to heat stress.

### Transcript profile clustering under MHS

**Figure 5:**
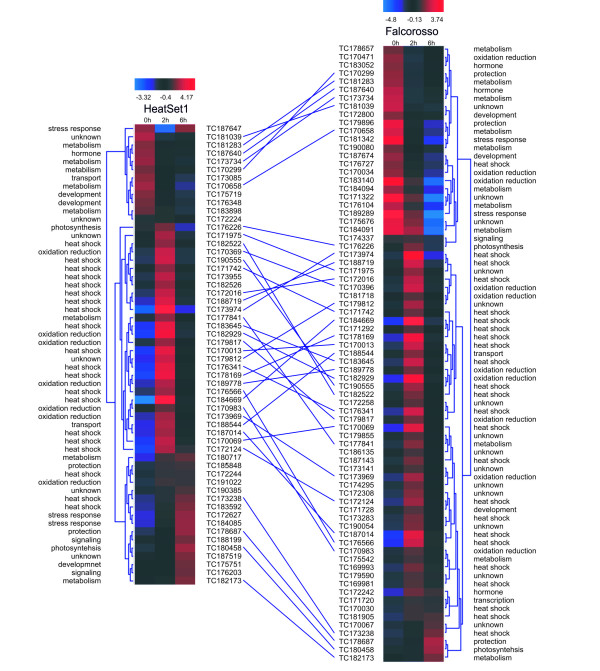
**Euclidian clustering of significantly differentially expressed genes in the heat-tolerant genotype (HS1) and the heat-sensitive genotype (FR)**. Heat maps of gene expression are shown with high expression shown in shades of red and low expression shown in blue. A dendogram of the expression profiles is shown to the left in HS1 (Heat Set 1) and the right in FR (Falcorosso). On either side of the heat maps, the TC number of the relevant probe is shown and the functional classification of the relevant gene. TCs common to both genotypes are indicated with lines linking the heat maps.

To analyse the gene expression profiles for each genotype, a Hierarchical Clustering (HCL) analysis was applied to the same set of 95 significantly differentially expressed genes also used in the O2PLS analysis (Figure 5). The wide transcriptional response in the heat-sensitive genotype (FR) also becomes clear with the difference between the numbers in both data sets. The largest cluster for both genotypes corresponds to a transient induction of gene expression activity, whereas up-regulation and down-regulation are the second most frequently occurring pattern in HS1 and FR respectively. The common probes in the different genotypes are indicated with a black line in Figure 5. This set has a largely similar expression profile between the two genotypes. 20 genes showing significant changes over the three time points are unique to the HS1 genotype whereas there are 39 unique differentials in the FR genotype (Figure 5). We conclude that HS1 shows a less extensive response in terms both of the intensity of transcriptional changes and the number of genes that are induced during MHS.

### Functional groups modulated by MHS

**Figure 6:**
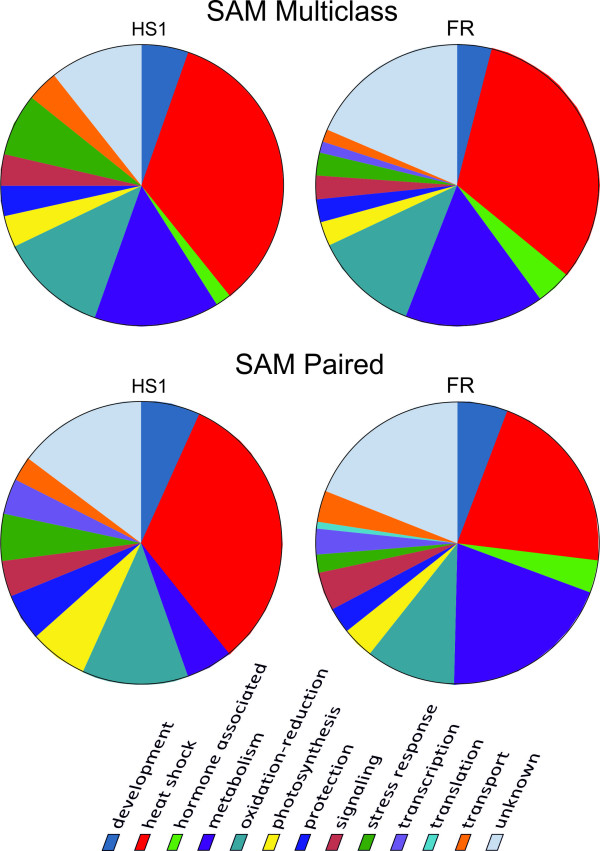
**Pie charts of the probable functional categorisation of the transcripts found to be significantly differentially expressed in the cDNA-AFLP and microarray experiment**. The two charts reflect the general (SAM multiclass) and in depth (SAM Paired) moderate temperature stress response in HS1, the heat-tolerant, and FR, the heat-sensitive genotype. The functional classes are colour coded.

The functional classification derived from DFCI gene index and UniProt annotation, illustrates the processes primarily affected in each of the genotypes. Genes were organized according to the metabolic processes in which they appear to be involved. Figure 6 shows the number of genes of each functional set illustrated as separate pie charts for the two genotypes and statistical analyses (SAM paired and multiclass). The multiclass SAM analysis of the stress response (upper section of Figure 6) reveals that the heat-tolerant and heat-sensitive genotypes react similarly, involving analogous functional groups: protection and repair, signalling and transcription, metabolism and development. Of the genes identified, the majority belongs to the protection and repair group (heat shock proteins, oxido-reductive molecules, stress response and protection) in both genotypes, while the metabolism and development group is much more highly represented in the heat-sensitive genotype and shows involvement of additional response pathways such as carbohydrate metabolism and hormone-related genes, according to the SAM paired analysis (lower part of Figure 6). By analogy, a transcriptomic analysis of tolerant and sensitive wheat strains indicates that transcripts coding for heat shock proteins, heat shock and other transcription factors are already turned on during acclimation for 3 h at 34° [35].

### Protection and repair transcripts represent the majority of genes affected by MHS

From the SAM paired analysis, the highest induced transcripts during the first 2 h of MHS in the tolerant genotype belong to the protection and repair genes and are similar to: AthHsp22.3 (TC185802, fold induction 1500), a class I heat shock protein (TC184669, fold induction 170), a class III sHsp (TC173974, fold induction 106), the cytosolic ascorbate peroxidase (TC182989, fold induction 36.6) and a class I sHsp (TC178169, fold induction 35.8). The highest induced transcripts in the sensitive genotype during the first two hours of MHS are a class I heat shock protein (TC184669, fold induction 73), a DNA-J (176514, fold induction 55), a cytosolic ascorbate peroxidase (TC182929, fold induction 34) and mitochondrial sHsp (TC187014, fold induction 32). However, the highest induction over the entire duration of MHS was recorded in both genotypes and consists of a similar group in each genotype: class I sHsp 17.6 (TC178169), class II sHsp 17.6 (TC170069), and mitochondrial sHsp (TC187014). Of these, only the class I sHsp 17.6 is more highly induced in the tolerant genotype (fold induction 36 as compared to 26 in FR). A single transcript corresponding to a DNA-J like protein (TC176727) was down-regulated in FR over the 6 h of MHS, but during the last 4 h of MHS, 15 heat shock proteins were down-regulated in expression in FR and 18 in HS1.

An additional set of genes that are differentially expressed exclusively in the heat-sensitive genotype, include heat shock proteins Hsa32 (TC191544), Rof1 (TC187143) and ROC7 (TC175253) genes that are required for protein folding. It is evident that oxido-reduction processes are affected by MHS in the heat-sensitive genotype from the differential expression of a carotenoid cleavage dioxygenase 1 (TC181718), a steroid alpha-reductase, (TC170471), a flavonol synthase (TC172800) and a flavoprotein (TC83140). The stress response of the heat-sensitive genotype can be seen from repression of transcripts similar to pepsin A (TC171322) and subtilisine (TC181342). Unlike the heat-sensitive genotype, the heat-tolerant genotype HS1 shows fewer genes significantly induced in the protection and repair group (Figure 6).

In conclusion, the heat-tolerant genotype HS1 reacts to MHS by enhancing the expression of heat shock proteins, oxido-reductive, transport and stress protective transcripts. In stark contrast, the heat-sensitive FR shows a more extensive, wider transcriptomic response characterised first by a complementary modulation pattern as compared to the heat-tolerant genotype. For example, transport and signalling genes are down-regulated in the heat-sensitive and up-regulated in the heat-tolerant. Secondly, the stress response in the heat-sensitive genotype is characterised by an amplified expression of a more diverse range of functional classes including transcription, photosynthesis, hormone related and protection genes which are increased, while transport and carbohydrate metabolism are reduced in expression. Thirdly, although the two genotypes involve similar functional classes in the response to heat, each genotype modulates "common" and "specific" genes, as described above. The genotype-specific modulated transcripts are of particular interest for the present work as their annotation may provide clues to the putative mechanism of heat tolerance.

The results obtained in the present study are similar to those obtained by Frank et al. [23], where the heat-tolerant genotype is shown to modulate fewer genes in response to heat, but also similar to results on other abiotic stresses [36-38]. The most marked differences between the two genotypes can be seen in the heat shock, metabolism and oxido-reduction groups: 32% (HS1) respectively 20% (FR) of the modulated genes belong to the heat shock group, 12% (HS1) respectively 20% (FR) belong to metabolic genes group and 8% (HS1) respectively 10% (FR) belong to the oxido-reduction group. It appears that the heat-tolerant genotype modulates mainly genes in the heat shock group while the heat-sensitive genotype modulates genes of the heat shock and metabolic groups in equal fractions; processes which may underpin the better performance of the heat-tolerant genotype under elevated temperatures.

### Metabolism and development transcripts are the second most affected by MHS

Interestingly, in comparison to the heat-tolerant genotype, all development-related genes significantly changed in expression by MHS were very highly expressed in the heat-sensitive genotype FR. In the metabolism group it is interesting to note that FR exhibits increased expression levels of carbohydrate metabolism genes as well, for example a transcript coding for beta-amylase (TC178310) is induced 3 fold in FR during the first 2 h of MHS but then it is repressed again. ß-amylase induction and the resultant maltose accumulation may function as a compatible-solute stabilizing factor in the chloroplast stroma in response to acute temperature stress [39].

### Transcription and signalling transcripts are also affected by MHS

Unlike the heat-tolerant genotype HS1, the heat-sensitive FR shows a different response with regard to transcription, translation and signalling. The ethylene-responsive transcriptional co-activator (TC171720) is transiently induced at 2 h by heat only in the sensitive genotype, which may also reflect the sensitivity of FR to MHS. It is known that the *A. thaliana *ortholog, AtMBF1c, enhances tolerance to heat and osmotic stress when over-expressed in *A. thaliana *[40]. Unlike the heat-tolerant genotype HS1, FR reacts with a different set of signalling partners and transcriptional regulators, down-regulating after 2 h of MHS a photoperiod responsive protein (TC177921) and an ARF5-like transcript (TC182088). In *Arabidopsis *hypocotyls, high temperature (29°C) causes an increase in free IAA, and this mediates a marked cell elongation response. Therefore, temperature signals may modify auxin synthesis or distribution in the plant, and this could represent a growth-regulating mechanism [41]. The heat-tolerant genotype reacts to heat by continuously increasing the expression of an Adagio 3-like transcript (TC188199) while another Bel1 transcriptional regulator (TC175335) is decreased at 6 h of MHS.

In addition, the heat-tolerant genotype not only reacted differently from the heat-sensitive during heat stress but also exhibited a constitutive and specific gene expression pattern, characterised by very high expression levels of protection and repair genes. The heat shock Hsp82 (TC170030) and the gene coding for mitochondrial small heat shock protein (msHsp) LeMtHSP (TC187014) are highly expressed in HS1 at time point 0 h prior to the MHS. Hsp82 is an hsp90 chaperone required for cell growth, is highly conserved among eukaryotes and the presence of at least one of the HSP90 gene product family members is essential for viability in yeast, *Drosophila*, and humans. In yeast, Hsp82 is part of the Hsp90 chaperone, which directly interacts with Hsf1p, the heat-shock transcription factor from yeast [42]. The reduction in tomato mitochondrial small hsp LEMTSHP expression, also known as Hsp23.8 [43] seems to lead directly to susceptibility to heat stress. In addition, a transcript similar to a cathepsin B-like cysteine proteinase (TC171192) is also constitutively higher expressed in the tolerant genotype. Cysteine proteinases have a positive role in plant growth, development, senescence and programmed cell death, but also in storage protein mobilization [44]. All these 3 genes are significantly increased after 2 h of MHS. The heat-tolerant genotype also exhibited a constitutive and specific gene expression pattern, characterised by differential expression levels of a carbohydrate metabolism gene, the fructose-1,6-bisphosphate aldolase (TC176475), which is highly expressed in HS1 at time point 0 h prior to the MHS. The fructose-1,6-bisphosphate aldolase is an enzyme of the glycolytic pathway. Reports from several laboratories have suggested that increased rates of glycolysis play an essential role in the initiation of DNA synthesis and may be involved in maintenance of replication and protein activity at high temperature [45].

It is interesting to note that in general, the heat-tolerant genotype HS1 appears to have an enhanced innate heat protection system exemplified by the hsp82 and hsp90 levels before and during the treatment. Furthermore, HS1 seems to have a lower rate of metabolism as exemplified by the low number of genes significantly changed in comparison to the sensitive genotype, not only before the treatment but also during the 6 h of MHS.

Unlike HS1, the sensitive genotype FR exhibits an increased number of genes (20) with a constitutively higher expression prior to the MHS, which may also indicate the sensitivity of this genotype to adverse conditions. For example, the highest constitutive expression in the sensitive genotype compared to the tolerant genotype is of genes that belong to the stress response pathway (TC189289; pepsin A, TC181342; subtilisin like protease), metabolism (TC176104; a transferase) and interestingly, in development (TC182608; histone H3, TC171121; male sterility 2 gene) or transcription (TC182088; ARF5). Of the genes constitutively higher expressed in the sensitive genotype, 19 are down-regulated within the first 2 h of MHS and only 1 is not affected at all by the MHS treatment (TC172148; an aquaporin involved in water and neutral solute transport).

The analysis of the stress response reveals that the tolerant and sensitive genotypes react largely by involving similar functional classes: heat shock, oxido-reduction, photosynthesis, stress response, protection, development, transcription and transport. The nature of the genes and intensity of their expression level is very genotype specific as has been described above. However, the group of transcript significantly changed in both genotypes shows probably the general response to heat and comprises in majority genes from the heat shock, metabolism and oxido-reduction functional classes.

The majority of "common" transcripts generally show the same induction pattern in both genotypes, but very often at different expression levels. However, the question whether they are relevant for tolerance is still open. For example, expression of a transcript similar to hsp 17.6 (TC170069), a class I shsp is higher and transiently induced at two hours of MHS in FR. A transcript similar to Hsc70 (TC176566) is also more highly expressed in FR as an result of heat. Other transcripts similar to Class I and Class III heat shock proteins are expressed more highly in the tolerant genotype in the first 2 h of stress. Oxidation and reduction genes are also induced over the first 2 h of heat and they are similarly induced in both genotypes, for example a cytosolic ascorbate peroxidase (TC170369); a central component of the reactive oxygen gene network in Arabidopsis [46], is more highly induced in the tolerant genotype than in the sensitive genotype. A metallothionein-like protein type 2 B (TC179817) involved in cellular response to stress [47] is expressed and transiently induced at a higher expression level in the sensitive genotype. TC170658, corresponding to a chalcone synthase 1 B involved in flavonoids biosynthesis, well known for its antioxidant properties [48], continuously decreases in expression in the two genotypes but the decrease is more pronounced in the sensitive genotype during the first 2 h of MHS. Photosynthesis is also affected, for example RUBISCO activase (TC176226) is equally increased at 2 h of MHS then decreased at the end of the heat treatment in both genotypes, however with a higher level in the sensitive genotype. Interestingly, expression of a transcript similar to the Zeatin O-glucosyltransferase ZOG (TC187640) decreases in the tolerant genotype over the entire duration of MHS but transiently decreasing in the sensitive genotype. The significance of this particular expression pattern may lie in the fact that ZOG regulates responses to water deficits and thus regulates stress protection by increasing active cytokinin levels [49].

It is clear that numerous biochemical pathways and metabolic routes are affected by MHS and the range of responses is generally broader in the sensitive genotype than in the tolerant. Thus it is not yet clear which genes are most directly involved in tolerance or in sensitivity to heat stress.

### Validation of microarray expression data

**Figure 7:**
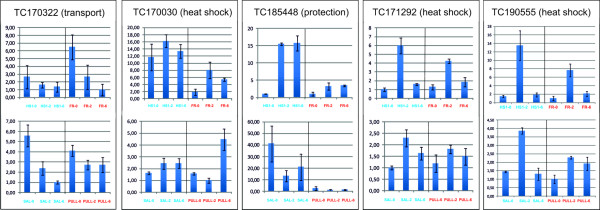
**Validation of the microarray profiling with q-PCR**. Candidate genes in heat-sensitive and heat-tolerant genotypes. Five transcripts corresponding to TC170030, TC171292, TC190555, TC185448 and TC170322 were verified with q-PCR in two pairs of contrasting genotypes: HS1 (heat-tolerant): FR(heat-sensitive), and Saladette (heat-tolerant): Pull (heat-sensitive), subjected to the same stress conditions at the same time points of 0 h, 2 h and 6 h of MHS.

In order to confirm the observed microarray expression profiles, q-PCR experiments were carried out. The expression pattern of five transcripts, from different functional classes was confirmed in the heat tolerant HS1 and the heat-sensitive FR genotypes, showing that heat have a clear influence on gene expression. Additional confirmation and correlation was obtained by performing q-PCR in another pair of contrasting genotypes: Saladette (heat-tolerant) and Pull (heat-sensitive). Transcripts coding for heat shock, transport and protection proteins show similar expression patterns to the ones observed from the SAM analyses. In addition, the second pair of contrasting genotypes showed a similar modulation in gene expression (Figure 7).

## Conclusions

Our transcript profiling experiments and those from others [23] demonstrate that many genes, involved in seemingly unrelated processes, are modulated by moderate heat stress. Expression of many of these genes is also modified in sensitive as well as in relative tolerant lines, differing only in amplitude. This suggests that tolerance is based on fine-tuning of quantitative expression of many genes. It is difficult to say whether the differences observed in our experiments are the basis of heat tolerance or solely a consequence of a better performing plant under stress conditions independent of a causative link. Furthermore, it is possible that different mechanisms exist in tomatoes, which have been selected during cultivation in different part of the world. The selection of tomato in cultivation is based on a small set of domestication genes causing little genetic variation in the tomato germplasm [50]. Based on this, an unintended selection during cultivation for epigenetic factors causing differential gene expression could partly explain differences in stress adaptation.

## Methods

### Plant material and heat stress conditions

**Figure 8:**
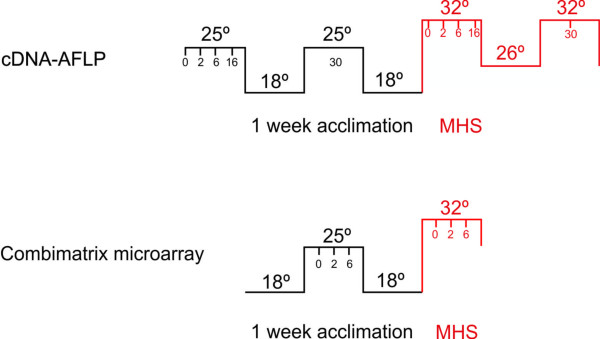
**Temperature regime for moderate heat stress (MHS) used in the cDNA-ALFP profiling (top) and in microarray experiment (bottom)**. Black lines represent the day/night temperatures prior to application of the MHS (acclimation). Sampling times are shown as hours after experimental onset below the temperature line. Red lines indicate the heat stress period with sampling times given as hours after onset of the MHS.

Three tomato heat heat-sensitive genotypes Moneymaker (MM), Falcorosso (FR), Pull and two heat heat-tolerant, Heat Set1 (HS1) and Saladette, were used. MM is a heat sensitive genotype according to our own observations. FR, obtained from Nunhems Netherlands BV (the Netherlands), is a commercial variety and HS1, also provided by Nunhems BV, is a variety selected for good fruit set in field conditions where temperatures are higher than 38°C. Pull and Saladette have been previously characterised as sensitive and tolerant genotypes [51]. Seeds were germinated in potting compost in trays and transferred after two weeks to a growth chamber under standard temperature (ST) conditions, with a 16-h light (26°C)/8-h dark (18°C) cycle. Fluorescent and incandescent lighting provided a photosynthetic photon flux density of 450-500 μmol m^-2 ^sec^-1^. Six-week-old plants were heat stressed by raising the temperature to a regime of MHS (32°C/26°C; day/night). The heat treatment was initiated by progressively increasing the temperature from 25 to 32° over half an hour period, and samples were collected at 0, 2, 6, 16 or 30 h of heat stress. In order to describe the dynamics of transcriptional responses of tomato developing meiotic anthers to MHS, the temperature range for the experiment was chosen based on agronomically relevant temperatures shown to have a significant effect on pollen viability [24], rather than using classical heat shock conditions of 42°-45°C [23]. Heat stress was applied to whole plants in the growth chamber according to the following scheme (Figure 8), under the same light conditions as stated above.

### Anther isolation and RNA extraction

We focused our analysis of gene expression on whole meiotic anther cones isolated from flower buds of plants that were exposed to MHS (32°C/26°C, day/night) for up to 30 h, and compared this to standard temperatures (26°C/18°C day/night). For the cDNA-AFLP and for the microarray experiment and in an attempt to reduce the biological variation, a pool of six meiotic flower buds from 3 plants were harvested (according to [52]). Pilot experiments indicated the third and fourth flower clusters to give the most consistent phenotypic response to heat stress (data not shown). From these, anther cones ranging in size from 2-4 mm were isolated, immediately frozen in liquid N_2 _and pooled for RNA extraction. Thus, tissues from pooled samples from 3 biological repeats were homogenized using glass rods and RNA was extracted using a Plant RNA isolation kit (Plant RNeasy Mini Kit, Qiagen Benelux BV, Venlo the Netherlands) according to the manufacturer's recommendations.

### Transcript profiling by cDNA-AFLP

For cDNA-AFLP, material was harvested at 0 h (ST) and at 2 h, 6 h, 16 h and 30 h from both ST and MHS treatments. The isolated RNA was subjected to cDNA-AFLP template preparation as described previously [25]. RNA fingerprinting was carried out using 92 primer combinations with 2 selective nucleotides and gave rise to an average of 80 transcript-derived fragments per primer combination (TDFs). Primer sequences were as described in Bachem et al. [25] (Additional file 2). The majority of bands showed no change in intensity in response to heat. Changes in the intensity of individual bands did not affect others in the same lane, indicating that product accumulation was not affected by the concentration of individual substrates in the reaction. Inconsistent bands were mainly observed in the region of the gel with very small DNA fragments (< 70 bp) and were therefore considered as aspecific amplification products. Bands corresponding to differentially expressed genes were cut out from the gel and the eluted DNA was re-amplified under the same conditions as for the selective amplification. Fragments were subsequently ligated in a T-tailed EcoRV digested phagemid (pBlueScriptII SK(+), Stratagene, La Jolla, CA, USA) and sequenced (CEQ™ DTCS Quick Start Kit and CEQ2000 DNA Analysis System, Beckman Coulter, Fullerton, CA, USA). Fragments that did not have the expected size, based on the height on the acryl amide gel from which they were isolated, were discarded. All cDNA-AFLP expression patterns displayed in the Results section, was confirmed by real-time quantitative PCR and using the microarray data.

### Microarray hybridization and data analysis

The 90 K Custom TomatoArray 1.0 chip (Combimatrix microarray platform, http://ddlab.sci.univr.it/FunctionalGenomics/) consists of 20200 unique probes derived from *Solanum lycopersicum *transcripts and various controls, and was produced by the Plant Functional Genomics Center, University of Verona. The gene specific probes (oligonucleotide of 35-40-mer) randomly distributed in quadruplicate across the array, were designed using the program design OligoArray 2.1 [53]. The sequences represented on the chip correspond to 20115 *S. lycopersicum *TCs (Gene Index Release 11.0, 21 June, 2006) and to 85 technical controls (negative and positive spiking controls). From the 20115 tomato probes represented on the chip, 17018 probes were found to be expressed above background and consistent among the technical repeats within each chip. The quality of the biological replicates was evaluated by Pearson coefficient, which ranged from 0.87 to 0.98. For the microarray experiment, 3 biological replicas for each sample were used. Material was harvested at 0 h (ST) and at 2 h and 6 h from both genotypes (FR and HS1). Amino allyl-RNA synthesis (aRNA) and labelling with the Alexa647 dye were performed from 1 μg of total RNA, with the "SuperScript TM Indirect RNA Amplification System" (Invitrogen, Carlsbad, CA) according to manufacturer's recommendations. Labelled aRNA was quantified by spectrophotometer and the efficiency of Cy5 dye incorporation (DOL) was calculated. 4 μg of labelled RNA, with DOL value ranging from 2.0 to 3.0, were first fragmented and hybridized to the array as indicated by the manufacturer (http://www.combimatrix.com) Pre-hybridization, hybridization, washings and imaging were performed according to the protocols given by CombiMatrix. The array was scanned with a ScanArray 4000 × L microarray scanner (Perkin Elmer, Waltham, MA USA). Tiff images were exported to the Microarray Imager 5.8 (Combimatrix) for the densitometry analysis of the spots.

The microarray data was normalized by median scaling and the quality of the biological replicates was assessed by means of Pearson's coefficient. Only those genes that were up or down-regulated at least twofold, relative to the average of the three negative controls, for at least one time point, were included in the analysis. The two-fold change in expression as threshold for consideration is a convention employed in various transcript profiling studies [23] and therefore is used here to assess the findings from the presented work in comparison with those of earlier reports. All microarray expression data are available at GEO under the series entry GSE24805. The resulting set of significantly changed expression values has been taken into further analysis with paired, unpaired and multiclass SAM analysis, to detect the effect of 'treatment' on expression levels. Genes with similar expression patterns were grouped according to a hierarchical clustering algorithm using Euclidean Distance coefficients.

The paired and unpaired SAM statistical analyses were carried out to determine the significant differences in gene expression between two time-points for each genotype and the significant differences in gene expression between individual time-points observed in both genotypes, respectively. These analyses were performed by T-Mev with a FDR = 5%. For each analysis, a new input dataset was created by filtering the data of the two samples that were compared on the basis of the C.V. values.

In order to determine the significant differences in gene expression accumulated over the entire duration of the experiment all data were filtered on the basis of the CV value (CV < 0.5) among spot replicates present on the chip and the restricted dataset obtained, of 17018 genes, was suited to run a multiclass comparison method of Significance Analysis of Microarray (SAM), with false discovery rate (FDR) = 5% [32].

The main components of the changes in gene expression in response to MHS were determined using the set of differentially expressed genes obtained by SAM multiclass analysis, by performing an O2PLS analysis with SIMCA P+ (Umetrics, Umea, Sweden). OPLS combines the existing theory of partial least squares (PLS) regression [54] and orthogonal signal correction (OSC) [55]. The unique property of O2PLS is its capacity to identify joint variation between two datasets, while acknowledging also systematic variation that is unique to each dataset. The expression profile clustering for each genotype was carried out with the Hierarchical Clustering (HCL) method with Euclidean Distance using T-Mev software (Version 4.3).

### Validation of gene expression by q-PCR

Total DNA-free RNA was isolated from tomato anthers (n > 5) using a RNA isolation kit (Qiagen RNeasy mini kit with the RNase-Free DNase Set, Qiagen). A PCR reaction with RNA- and DNA-specific primers (Additional file 2) based on an intron in the *S. lycopersicum *actin gene Tom51 (GenBank accession number U60481) was performed to ensure the absence of contaminating genomic DNA. Total DNA-free RNA (1 μg) was used for cDNA synthesis (iScript™ cDNA Synthesis Kit; Bio-Rad Laboratories, Hercules, CA, USA) in a total volume of 25 μl. PCR reactions were carried out in 25 μl containing 0.125 μl of cDNA synthesis reaction mixture, 400 nm of each primer and 12.5 μl of iQ SYBR Green Supermix (Bio-Rad Laboratories). PCRs were performed in a 96-well Bio-Rad iCycler (Bio-Rad Laboratories) using a temperature program starting with 3 min at 95°C followed by 40 cycles consisting of 15 s at 95°C and 45 s at 57°C, and finally the melting temperature of the amplified product was determined to verify the presence of a specific product. Fold changes in expression levels were calculated using the MS-Excel macros (Biorad) where the lowest expression value was set to one.

In addition, a fraction of the PCR mixture was analysed on a 1% agarose/ethidium bromide gel to check the size of the amplified DNA fragment. The primers that were used for the real-time quantitative PCR reactions were designed using a computer program (Beacon Designer 5.01; Premier Biosoft International, Palo Alto, CA, USA) to obtain primers that have close to identical melting temperatures and do not form secondary structures with each other in the given PCR conditions. Primer sequences are listed in Additional file 2. In addition, to enhance primer efficiency, primer-binding sites were chosen such that secondary structures of the template were avoided. All reactions were performed on a pool of 6 anther cones collected from 3 different plants. The pooling has been done in such a way as to minimize the eventual biological variation.

### Functional classification criteria

For the microarray analysis, the 20115 tomato probes were designed from sequences deposited in the tomato TGI database [56]. Thus, the annotations of the gene sets on the cDNA-AFLP or on the microarray were taken from the DFCI Tomato Gene Index (LGI), which integrates research data from all international tomato gene research projects. Where biological ontology was not clear, the UniProt Knowledgebase provided further functional information.

### Accession numbers

All microarray expression data are available at GEO under the series entry GSE24805.

## List of abbreviations

cDNA-AFLP^®^: cDNA Amplified Fragment Length Polymorphism.

## Authors' contributions

***CEB and WV ***carried out the molecular genetic studies, participated in the microarray analysis and wrote the manuscript. SZ and MP performed the statistical analysis. CM and TG helped to draft the manuscript.

All the authors have read and approved the final manuscript.

## Supplementary Material

Additional file 1 **Details of probes on the microarray that showed significantly changed gene expression**. Probe details (homologies, expression patterns in the genotypes and functional classification) arising from the SAM statistical analysis (SAM multiclass, SAM paired, SAM unpaired) Click here for file

Additional file 2 **Q-R/T PCR primers**. Names and sequences of PCR primers used in the quantitative R/T PCRs to verify gene expression levels found in the high-through-put expression profiling. Click here for file
